# Laparoscopic Suture versus Mesh Rectopexy for the Treatment of Persistent Complete Rectal Prolapse in Children: A Comparative Randomized Study

**DOI:** 10.1155/2020/3057528

**Published:** 2020-01-22

**Authors:** AbdelAziz Yehya, Ibrahim Gamaan, Mohamed Abdelrazek, Mohamed Shahin, Ashraf Seddek, Mohamed Abdelhafez

**Affiliations:** Pediatric Surgery Department, Al-Azhar University Hospitals, Cairo, Egypt

## Abstract

**Purpose:**

To compare laparoscopic mesh rectopexy with laparoscopic suture rectopexy. *Patients and Methods*. The prospective study was conducted at Pediatric Surgery Department, Al-Azhar University Hospitals, Cairo, Egypt between Feb 2010 and Jan 2015. Seventy-eight children with persistent complete rectal prolapse were subjected to laparoscopic rectopexy. Fourteen parents refused to participate. All patients received initial conservative treatment for more than one year. The remaining 64 patients were randomized divided into two equal groups. Group A; 32 patients underwent laparoscopic mesh rectopexy and group B, 32 underwent laparoscopic suture rectopexy. The operative time, recurrence rate, post-operative constipation, and effect on fecal incontinence, were reported and evaluated for each group.

**Results:**

Sixty-four cases presented with persistent complete rectal prolapse were the material of this study. They were 40 males and 24 females. Mean age at operation was 8 (5–12) years. All cases were completed laparoscopically. Mean operative time in laparoscopic suture rectopexy was shorter than laparoscopic mesh rectopexy group. No early post-operative complications were encountered. No cases of recurrence with mesh rectopexy group while in suture rectopexy group it was 4 cases (14.2%). Post-operative constipation occurred in one case (3.57%) in suture rectopexy group and occurred in one case (3.3%) in mesh rectopexy group. Fecal incontinence improved in 26/28 cases (92.8%) in suture rectopexy while in mesh rectopexy it was improved in 30/30 cases (100%) of cases.

**Conclusion:**

Both laparoscopic mesh and suture rectopexy are feasible and reliable methods for the treatment of complete rectal prolapse in children. However, no recurrence, low incidence of constipation and high improvement of incontinence at follow up more than 36 months with mesh rectopexy accordingly, we considered mesh rectopexy to be the procedure of choice in treatment of complete rectal prolapse.

## 1. Introduction

Rectal prolapse is a relatively common condition in young children. The peak incidence is between 1 and 3 years of age. It is more common in children younger than 4 years of age with male-to-female ratio about 2-3 : 1. It mostly has no identifiable underlying cause, but constipation is reputed to play a role by stretching and exhausting pelvic floor and anal sphincter muscles. It can range from mucosal prolapse to full-thickness prolapse, which requires manual reduction [1]. Predisposing factors include more vertical course and less prominent curves of the rectum, more flat sacrum and coccyx, a relatively low position of the rectum in the pelvis, and poor levator muscle support [2]. It is usually self-limited & cases respond to conservative measures. However, occasionally surgery is recommended for full-thickness persistent prolapse, particularly in children with associated disease [3]. Patients are treated for the underlying cause such as chronic constipation or acute diarrhea and they should avoid straining on the toilet. Generally, surgical intervention is not recommended until 1 year of conservative therapy. However, this period is adjusted depending on severity of prolapse and associated stress on the patient and family [4]. Repair of complete rectal prolapse depends on fixation of the rectum against the presacral fascia [5]. Controversy exist about the optimum corrective surgery as there are many surgical options described including submucosal injection of sclerosants, Thiersch cerclage, abdominal rectopexy with or without sigmoid resection, posterior sagittal rectopexy, and perineal rectopexy [6]. Recently many reports address the success of different minimally invasive laparoscopic approaches for complete rectal prolapse, namely, suture rectopexy, posterior mesh rectopexy, while resection of the sigmoid colon, with or without rectopexy, is reserved for intractable constipation with redundant rectosigmid to shorten the colon and reduce total colonic transit time [7].

## 2. Patients & Methods

This prospective comparative randomized study was conducted on 64 patients with complete rectal prolapse at Pediatric Surgery Department, Al-Azhar University Hospitals, Cairo, Egypt in the period between Feb 2010 and Jan 2015 were listed and evaluated. With the approval of our institutional ethical committee, written informed consents were obtained from all patients to be included in this study, after explanation of disease nature and different methods of treatment. Randomization was achieved through sealed envelopes that opened in the operating room. Then the 64 patients were randomly divided into two groups. Group A, 32 patients underwent laparoscopic mesh rectopexy and group B; 32 patients underwent laparoscopic suture rectopexy.

**Figure 1:**
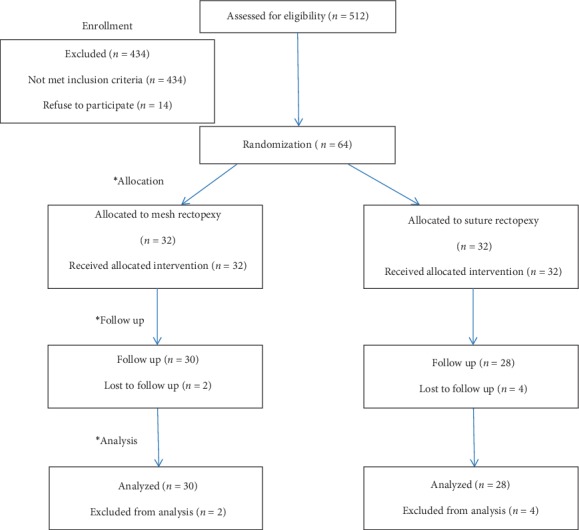
Algorithm of randomization trail. (i.e., enrollment, allocation, follow up and analysis of data).

Inclusion criteria included full-thickness persistent rectal prolapse after failure of proper conservative treatment, frequent prolapse >2 episodes requiring manual reduction under sedation, and associated rectal pain and bleeding. Exclusion criteria case associated with intractable constipation (infrequent, painful defecation or both for 2 weeks or more and sufficient to cause child distress) confirmed by barium enema or prolonged total colonic transit time, extensive adhesions from prior abdominal surgery, cases older than 14 years old, and cases of rectal polyps (secondary rectal prolapse). All patients received an initial period of conservative treatment in the form of (16 cases had previously undergone single cerclage, 26 cases had twice cerclage, 12 cases had injection sclerotherapy and 10 cases consecutive injection then cerclage). A decision to operate is based on age of the patients, duration of conservative management and frequency of recurrent prolapse >2 episodes requiring manual reduction along with symptoms of pain and rectal bleeding. They were subjected to thorough clinical assessment, routine laboratory investigations including stool analysis, plain radiography, Barium enemas, sigmoidoscopy in cases with bleeding per rectum, and pre- and postoperative (6 months later) electromyography [EMG] activities for external anal sphincter and puborectalis muscles during rest, on minimal volition and maximal squeeze. The main outcome parameters of the study included; the operative time, recurrence rate, post-operative constipation, and effect on fecal incontinence. Pre-and post-operative assessment of fecal incontinence was done using the Wexner score (Table 1 and Figure 1).

### 2.1. Operative Technique

Each patient underwent colonic preparation by twice warm saline enema (one at night before surgery & one at the morning of surgery) and received one dose of cefotaxime, 50 mg/kg, and metronidazole, 7.5 mg/kg in the morning of surgery. In both groups, after induction of general end tracheal tube anesthesia, a uretheral catheter was inserted and the patient was placed supine in Trendelenburg's position. The surgeon and scrub nurse stand on the right side while the assistant surgeon stands on the left side of the patient. A 5 mm trocar was placed above the umbilicus by open method was done, then pneumoperitoneum was created to a pressure of 8–12 mmHg· 5 mm 30 scope was introduced through the umbilical port to visualize the abdomen and pelvis. Two 3-mm accessory trocars (one at each lateral quadrant at or below the level of umbilicus according to child's age & body built) were placed via stab incision under direct vision for insertion of working instruments. The redundant rectosigmoid was delivered from the pelvis using a traumatic grasper. Laparoscopic exploration started and by assessment and manipulation of rectosigmoid redundancy in all cases. A percutaneous suture was inserted through the seromuscular layer of sigmoid colon for traction on the rectosigmoid. The peritoneum over the sacral promontory on right side of the rectum was incised using hook electrocautry or a scissors, extending to the posterior rectal wall with great care to save to the mesorectum, the ureter and external iliac vessels to enter the retrorectal space and presacral fascia with preservation of the lateral rectal ligament.

This right side approach was preferred for easy manipulation with a good visualization and proper exposure of the retrorectal space with elevation and traction of the anterior wall of the rectum anteriorly laterally and towards the left side with left hand Grasper allowing adequate plane of dissection between the posterior wall of the rectum and the sacrum both safely and rapidly (right handed surgeon).

Both laparoscopic mesh rectopexy and laparoscopic suture rectopexy include retrorectal dissection from level of sacral promonatry to the pelvic floor 3-4 cm above the dentate line. In suture rectopexy the posterolateral rectal wall was sutured to the periosteum of sacral promontory and presacral fascia using non-absorbable suture (proline 2\0) in two seromuscular bites 2 cm apart on right side of the rectum.

Mesh rectopexy was done by insertion of polypropylene (Proline)mesh measuring about (7 × 2 cm) tailored, inserted and fixed to the middle of the posterior wall of the rectum and presacral fascia using praline 2\0. At the end of any of the two techniques, the peritoneal defect was closed by continuous absorbable (Vicryl 2/0) suture.

Posterior mesh rectopexy was done for all cases of group A by the same surgeon, who had good experience about posterior approach not for anterior approach.

At the end of the procedure, the rectum extends straight in the pelvis with a mild tension. The abdomen was deflated and the port sites were closed with absorbable suture (Polygalactin–Vicryl–2/0).

Early oral intake was advised as most enhanced recovery programs concluded by fluid intake within a few hours of surgery.

Post-operatively, patients were discharged home on about the 2^nd^ postoperative day on acetaminophen and or NSAID for pain/discomfort and advised to avoid straining & constipation by keeping on high-fiber diet, plentiful fluids, and laxatives as lactulose.

All patients were followed up at the outpatient clinic by contributing authors responsible for data collection with double blinded observation using a formal questionnaire. They examined the patients in sitting position 1 week postoperatively for clinical assessment of recurrence, degree of continence and constipation, then 1, 3, 6 months after operation and then every year for three years. EMG external anal sphincter & pelvic floor muscles were repeated months later, then if indicated.

## 3. Statistical Methods

### 3.1. Sample Size Justification

MedCalc® version 12.3.0.0 “Ostend Belgium” program was used for calculations of sample size, statistical calculator based on 95% confidence interval and power of the study 80% with *α* error 5%, sample size was calculated according to these values produced a minimal samples size of 25 cases in each group.

### 3.2. Statistical Analysis

The collected data will be, tabulated, and statistically analyzed using SPSS program (Statistical Package for Social Sciences) software version 20.0 (SPSS Inc., Chicago, Illinois, USA). Inferential analyses were done for qualitative data using Chi square test for independent groups. The level of significance was taken at *P* value <0.050 is significant, otherwise it is not significant. The *p*-value is a statistical measure for the probability that the results observed in a study could have occurred by chance.

## 4. Results

From Feb 2010 to Jan 2015, sixty-four children with complete persistent rectal prolapse were operated upon laparoscopic ally for repair. Mean age at the time of operation was 8 years (range 5–12 years). They were 40 males (62.5%) and 24 females (37.5%) with M : F ratio of 1.6 : 1. Mean duration of symptoms was 2.9 ± 1.8 years (range 5 months–7 years). Both groups were comparable in demographic data and preoperative clinical presentation. In all cases a constant laparoscopic findings were present in the form of mild peritoneal reaction, redundant rectosigmoid and hypertrophy of mesenteric lymph node. All procedures were completed laparoscopically without conversion. Blood loss was limited, controllable in all cases. The mean operative time was 45.7 minutes (range 34–60) for laparoscopic suture rectopexy, whereas for laparoscopic mesh rectopexy it was 62.05 minutes (range 35–105). No intra-operative complications occurred in this study. Oral intake was resumed within a mean period of 24.3 hours (range, 20–30) after suture rectopexy & 27.3 hours (range, 22–35) after mesh rectopexy. Mean hospital stay was 2.5 days (range 2–5) for suture rectopexy and it was 2.4 days (range 2–4) for mesh rectopexy. No early post-operative complications such as port-site infection, port-site hematoma or hernia, bowel obstruction, pelvic collection, or fecal impaction had ever occurred. Six cases were lost to follow up. The remaining 58 patients were 30 cases in group A, and 28 cases in group B (Table 2). The patients were available for follow-up for a mean period of 36  months (range 30–42 months) after laparoscopic suture rectopexy, and for a mean period of 40 months (range 30–50 months) after laparoscopic mesh rectopexy. Sigmoidoscopy was done to patients presented with bleeding per rectum (all normal). Barium enemas were also done in few cases with no patients having other pathology. One patient presented with ectopia vesica not included in our cases. No cases of recurrence in group A, while in group B the recurrence occurred in 4 cases (14.2%). One case (3.57%) developed new onset constipation post-operatively after suture rectopexy compared to one case (3.3%) of occasional constipation after mesh rectopexy (Table 3). All cases of constipation respond well to conservative treatment. No side effects of mesh such as erosion, fistulation, migration, or infection have occurred. By application of Waxener score and according to EMG status, 56/58 (96.5%) cases with improved continent from grade 3 and 4 to grade 1; 26/28 (92.8%) within the suture rectopexy group while 30/30 (100%) belonged to the mesh rectopexy group. None of our patients had cystic fibrosis.

## 5. Discussion

Our study had a large number of cases indicated for surgical interference along the five years period, referred to our university hospitals draining the overcrowded wide area also, rectal prolapse is more common in Egypt due to malnutrition and parasitic infestation.

Rectal prolapse (RP) is an entity commonly seen in the pediatric population, encountered most often between 1 and 3 years of age. The etiology in children is usually idiopathic. Often in the setting of toilet training when parents encourage prolonged times on the commode [8].

In children it is usually a self-limited problem so its management is initially conservative [9, 10]. In a small number of cases, particularly those presenting after 4 years of age, the problem is persistent and causes a variety of symptoms including bleeding, tenesmus, and pain [11].

Randall et al. [12] stated that less than 10% of rectal prolapse cases require a surgical treatment, while Koivusalo et al. declared that the ratio is about 14%.

Cystic fibrosis is associated with nearly a 20% incidence of rectal prolapse in some reports, but most children with prolapse do not have diagnosed CF, particularly in the absence of symptoms [13].

None of the current study patients neither had clinical picture of cystic fibrosis nor do they belong to its geographical distribution area.

In our study, one case has been associated with recurrent ectopia vesica and this compares with a similar association reported by Koivusalo et al. and one case of our study developed complete rectal prolapse after Soave endorectal pull-through and this compares with a similar sequel reported by Potter et al.

In our study, idiopathic rectal prolapse was present in 34 cases (53%); in comparison to 9 of 19 cases (47%) reported by Potter et al.

Preoperative constipation was present in 22 cases (34.3%) of our study patients and this is nearly the same as reported by Potter et al. (32%, 6 of 19 cases) and compares with the ratio of 27% (3 of 11 cases) reported by Randall et al. This ratio also lies within the range (3–53%) reported by other authors [2, 11, 13].

Surgery for persistent rectal prolapse is rarely required in children. Thus, the ideal procedure should be minimally invasive and have a low recurrence rate and minimal functional consequences on the bowel, mainly on constipation [14].

The specific goals of surgical management of full-thickness rectal prolapse are to restore the anatomy & physiology, by control of the external prolapse of the rectum, improve continence, improve bowel function (prevent constipation or impaired evacuation), and to reduce the incidence of recurrence with lower morbidity & mortality [15].

The laparoscopic approach has the advantages of early recovery, less blood loss, less pain medication, favorable pain and mobility scores, but with a longer operating time [16]. Both laparoscopic resection rectopexy and laparoscopic suture rectopexy without resection have been described, each with its own merits [17].

Laparoscopic colorectal resection with rectopexy is preferable in patients with history of intractable constipation and prolonged total colonic transit time because it is unwise to fix the rectum by any means against chronic straining in those cases [18].

Laparoscopic full posterior rectal mobilization and fixation to the sacrum alone has a high success rate, lower mortality and morbidity and has a lower risk of sepsis & recurrence [19].

In our study, a 5-mm umbilical port was used for scope insertion. This compares with 10-mm umbilical port utilized by Koivusalo et al. A percutaneous rectosigmoid colonic traction suture placed at the left lower quadrant saved an additional fourth port that was added by other authors for the same purpose.

Unilateral right-side pararectal dissection was done in all cases of the study and none underwent bilateral dissection to avoid exposing both ureters and external iliac vessels to any risk of injury during the procedure. This is in contrast to bilateral pararectal dissection described by Puri and circumferential (complete perirectal) dissection described by Gomes-Ferreira et al.

Other authors as Ismail et al., Shalaby et al. & Gomes-Ferreira et al. described this step. Polypropylene (Prolene) suture was utilized in all cases of our study in comparison to silk suture utilized by other authors as Koivusalo et al., Laituri et al., & Puri. Significant pelvic sepsis is a major contributor to the postoperative morbidity, having been reported in 2–16% of patients with prosthetic (mesh) rectopexy [20].

In our study, no sepsis had been encountered in any case of the laparoscopic mesh rectopexy as all patients received antibiotic prophylaxis in the form of cefotaxime. Careful retrorectal dissection to avoid pelvic hematoma formation and avoidance of post-operative peritoneal drainage might be other protective factors.

In our study, the mean operative time was 45.7 minutes (range 34–60 minutes) for suture rectopexy and this compares well with Koivusalo et al. who reported a mean operative time of 80 minutes and Ismail et al. who reported a mean of 60 minutes. Potter et al. reported a mean of 72 minutes, while Puri reported a mean of 75 minutes. Whereas the mean operative time for laparoscopic mesh rectopexy was 62.05 minutes (range 35–105) and this is comparable with other studies as Ismail et al. who reported a mean operative time of 90 minutes and Shalaby et al. who reported a mean of 40 minutes. Also, Gomes-Ferreira et al. reported a mean of 98 minutes for laparoscopic modified Orr-Loygue mesh rectopexy.

Lateral rectal ligaments (rectal stalks) were preserved in all cases of our study. It appears that patients have less constipation and incontinence if the lateral rectal ligaments can be preserved; however, this requires further analysis [21].

Oral intake was resumed in our study within a mean period of 24.3 hours (range 20–30) after suture rectopexy & 27.3 hours (range 22–35) after mesh rectopexy. Length of hospital stay is an indicator of the patient's recovery and post-operative complications.

In our study, it is shorter or the same as the published ones.

One of the important parameters to gauge the success of rectal prolapse surgery is the rate of recurrence. Recurrence after suture rectopexy has been reported as ranging from 0% to 3% [22]. This compares with a recurrence rate of 0% reported by Koivusalo et al., Laituri et al. & Ismail et al. Also this compares with a rate of 5.5% full-thickness recurrence & 11% partial recurrence reported by Potter et al. 5% partial (mucosal) recurrence reported by Puri. Randall et al. have reported a strange very high disappointing failure rate with laparoscopic suture rectopexy [100% of their cases (*n* = 5)].

Benoist et al. published their results of laparoscopic rectopexy in 48 patients. They evaluated laparoscopic rectopexy using mesh, suture and resection and concluded that there was no difference among the three groups in terms of continence and recurrence and that mesh rectopexy conferred no advantage over suture rectopexy [23]. This compares with 0% recurrence rate reported by Ismail et al. & Shalaby et al., and 0–5% reported by Gomes-Ferreira et al.

In our study, two cases experienced post-operative constipation one case (3.57%) reported with laparoscopic suture rectopexy and one case (3.3%) followed laparoscopic mesh rectopexy. This compares with Koivusalo et al. who found post-operative constipation in 2 of 8 cases (25%) after laparoscopic suture rectopexy compared to 1 case after posterior sagittal rectopexy (PSRP). Ismail et al. reported 1 case of 40 (2.5%) developed post-operative constipation. Shalaby et al. found 1 case of 52 (1.9%) with post-operative constipation. Similarly, Puri reported 1 case of 19 (5.3%) with post-operative constipation. On the contrary, Gomes-Ferreira et al. reported zero percent post-operative constipation in their series. It has been speculated that loss of compliance of rectum after rectopexy or a redundant sigmoid loop may be associated with constipation [24].

In suture rectopexy technique, the lateral rectal ligaments were not divided. Reports concerning the advantages of preservation of the lateral rectal ligaments are conflicting. It appears that patients have less constipation and incontinence if the ligaments can be preserved [25]. This may be specially so because the lateral ligaments containing the parasympathetic inflow to the left colon may be cut during mobilization. At least two studies have demonstrated a higher incidence of constipation with significant changes in rectal sensation when lateral ligaments are divided as compared with when they are not [26].

Suture rectopexy has been shown to be equally effective as mesh rectopexy in preventing recurrence but avoids the problems of post-operative sepsis and increased constipation [27].

In a randomized clinical study that compared Wells mesh rectopexy with simple suture rectopexy, the results confirmed equal anatomical results, and better functional results (less constipation) and fewer complications with the simple suture [28].

In a series of 42 patients of complete rectal prolapse undergoing suture rectopexy, Rose et al. reported that 90% patients were found to have improvement in the continence levels post-operatively [29].

In our series, the low number of children with postoperative constipation and recurrent of prolapse may be attributed to the routine correction of rectosigmoid redundancy by left lateral fixation which put it in a good alignment without any angulations and prevents intussusceptions.

Strength of this study is that both groups of patients have balanced demographic data and preoperative clinical presentation, length of follow up, only two surgeons performed all procedures and 91.75% of patients had objective follow up and precise assessment by EMG.

The limitations of present study are that it was a single center experience, the median follow up time was rather short, and also we need a large randomized study to assess and support the value of both techniques and compare them with other approaches.

## 6. Conclusion

Both laparoscopic mesh rectopexy and suture rectopexy are feasible and reliable methods for the treatment of complete rectal prolapse in children. However, no recurrence, low incidence of postoperative constipation and good result improvement of incontinent at follow up more than 36 months with mesh rectopexy. Accordingly, we considered mesh rectopexy to be the procedure of choice in treatment of complete rectal prolapse.

## Figures and Tables

**Table 1:** 

Type of incontinence	Never	Rarely	Sometimes	Usually	Always
Solid	0	1	2	3	4
Liquid	0	1	2	3	4
Gas	0	1	2	3	4
Wears pad	0	1	2	3	4
Life style alteration	0	1	2	3	4

^∗^Never, 0; rarely, <1/month; sometimes, <1/week, >1/month; usually, <1/day, >1/week, Always. >1/day

^∗^0 :  perfect; 20 : complete incontinence.

**Table 2:** Demographic data in both groups.

Characteristics	Group A (mesh rectopexy)	Group B (suture rectopexy)
*Number of patients*	30	28
Male	19	17
Female	11	11

*Symptoms*		
Prolapse	30	28
Bleeding per rectum	24	22
Pain	3	3

**Table 3:** Follow up results in both groups.

Variables	Group A (mesh rectopexy)	Group B (suture rectopexy)	*P*-value
Hospital stay	2.4 days (2–4)	2.5 days (2–5)	NS
Operative time	(30–105) 62.5 min	(34–60) 45.1 min	NS
Mean follow up	40 (30–50) months	36 (30–42) months	NS
Recurrence	0/30 (0%)	4/28 (14.2%)	0.038
Constipation	1/30 (3.3%)	1/28 (3.57%)	NS
Continence	30/30 (100%)	26/28 (92.8%)	NS

## Data Availability

Data are not available to other researchers because they are from a registry or institutional database of patients providing routinely collected data.
